# Graded perturbations of metabolism in multiple regions of human brain in Alzheimer's disease: Snapshot of a pervasive metabolic disorder

**DOI:** 10.1016/j.bbadis.2016.03.001

**Published:** 2016-06

**Authors:** Jingshu Xu, Paul Begley, Stephanie J. Church, Stefano Patassini, Katherine A. Hollywood, Mia Jüllig, Maurice A. Curtis, Henry J. Waldvogel, Richard L.M. Faull, Richard D. Unwin, Garth J.S. Cooper

**Affiliations:** a School of Biological Sciences, Faculty of Science and Maurice Wilkins Centre for Molecular Biodiscovery, University of Auckland, Auckland, New Zealand; b Institute of Human Development, Faculty of Medical and Human Sciences, University of Manchester, Manchester, UK; c Auckland Science Analytical Services, Faculty of Science, University of Auckland, Auckland, New Zealand; d Centre for Brain Research, Faculty of Medical and Health Sciences, University of Auckland, Auckland, New Zealand; e Centre for Advanced Discovery and Experimental Therapeutics (CADET), Central Manchester University Hospitals NHS Foundation Trust, Manchester Academic Health Sciences Centre, Manchester, UK; f Department of Pharmacology, Medical Sciences Division, University of Oxford, Oxford, UK

**Keywords:** Alzheimer's disease, Neurodegeneration, Metabolic disorder, Metabolomics, Gas chromatography–mass spectrometry, Brain amino-acid metabolism

## Abstract

Alzheimer's disease (AD) is an age-related neurodegenerative disorder that displays pathological characteristics including senile plaques and neurofibrillary tangles. Metabolic defects are also present in AD-brain: for example, signs of deficient cerebral glucose uptake may occur decades before onset of cognitive dysfunction and tissue damage. There have been few systematic studies of the metabolite content of AD human brain, possibly due to scarcity of high-quality brain tissue and/or lack of reliable experimental methodologies. Here we sought to: 1) elucidate the molecular basis of metabolic defects in human AD-brain; and 2) identify endogenous metabolites that might guide new approaches for therapeutic intervention, diagnosis or monitoring of AD. Brains were obtained from nine cases with confirmed clinical/neuropathological AD and nine controls matched for age, sex and *post-mortem* delay. Metabolite levels were measured in *post-mortem* tissue from seven regions: three that undergo severe neuronal damage (hippocampus, entorhinal cortex and middle-temporal gyrus); three less severely affected (cingulate gyrus, sensory cortex and motor cortex); and one (cerebellum) that is relatively spared. We report a total of 55 metabolites that were altered in at least one AD-brain region, with different regions showing alterations in between 16 and 33 metabolites. Overall, we detected prominent global alterations in metabolites from several pathways involved in glucose clearance/utilization, the urea cycle, and amino-acid metabolism. The finding that potentially toxigenic molecular perturbations are widespread throughout all brain regions including the cerebellum is consistent with a global brain disease process rather than a localized effect of AD on regional brain metabolism.

## Highlights

• Impaired brain metabolism is a key pathogenic property of sporadic Alzheimer's
• 69 altered metabolites were profiled in 7 brain regions in this case–control study.
• Severe regulatory defects in energy-producing and amino-acid pathways, and urea
• First metabolic profiling study of region-specific alterations in human AD brain
• Compelling evidence that sporadic Alzheimer's is a metabolic brain disease

## 1 Introduction

Alzheimer's disease (AD) is the most common cause of dementia and is clinically characterized by a progression from episodic memory problems to a slow general decline of cognitive function [1]. In 2013, ~ 44 million of the world-wide population was estimated to be affected by dementia and a steep rise to ~ 136 million has been predicted by 2050 [2]. To date, there are no treatments with proven disease-modifying effects and AD remains the largest unmet medical need in neurology [1].

AD pathology presents a complex interplay between several biochemical alterations, including changes in amyloid precursor protein metabolism, phosphorylation of the tau protein, oxidative stress, impaired energetics, mitochondrial dysfunction, inflammation, membrane lipid dysregulation and neurotransmitter pathway disruption [3]. Most of these pathological features can be directly linked to metabolic abnormalities and it is now clear that metabolic dysfunction is an important factor in AD [4]. For example, impaired cerebral glucose uptake occurs decades prior to the onset of cognitive dysfunction and is an invariant feature of AD [5]. The well-documented neurotoxicity associated with Aβ42 is thought to participate in impaired neuronal energetics through initiating a cascade of pathological events; interaction between Aβ42 and mitochondrial enzymes leads to increased release of reactive oxygen species (ROS), affecting glycolysis, the TCA cycle and mitochondrial respiratory-chain activity through the accumulation of deleterious intermediate metabolites in the mitochondria [6], [7].

As the brain performs diverse functions, ranging from motor-sensory to behavioral and cognitive regulation [8], a systematic examination of metabolites is required to elucidate both the diversity and specificity of metabolic processes, and their alterations in the brain during disease processes such as in AD.

Gas-chromatography mass-spectrometry (GC–MS) is one of the most frequently used metabolite profiling tools and has previously been applied to serum samples from AD patients. Trimethylsilyl derivatives [9] have seen widespread application in a range of biological studies. The method applied here is based on an “untargeted metabolomics” procedure which gives broad coverage of a range of common metabolites; it generates reliable measures of relative metabolite levels in groups under comparison.

To our knowledge, there has not been a global GC–MS based metabolite profiling of the human AD-brain reported to date. This may partly be due to technical challenges in optimizing a GC–MS platform suitable for such analysis. Another likely reason is the substantial difficulties in acquiring high-quality human brain tissue required for such studies. Researchers have suggested that lack of high-quality translational research on human brain tissue is a major limiting factor in progressing drug design for disease-modification in AD [10]. Current FDA-approved drugs for AD are based on findings from *post-mortem* human brain studies performed more than three decades ago [10].

The Human Brain Bank (HBB) at the Centre of Brain Research (New Zealand) provides high-quality, well-characterized brain tissue collected under well-controlled conditions [11]. We here report our systematic examination of changes in metabolites in *post-mortem* brains from patients with AD compared to age- and sex-matched controls with no clinical dementia, using a previously-published, validated GC–MS-based methodology [12]. Since some brain regions are more heavily affected by AD than others [13], we compared and contrasted results from seven functionally-distinct regions that are considered to be *severely affected*: hippocampus (HP), entorhinal cortex (ENT), and middle-temporal gyrus (MTG) [13], [14]; *moderately affected*: sensory cortex (SCX), motor cortex (MCX), and cingulate gyrus (CG); along with one control region, cerebellum (CB), which is believed to be *relatively spared* [15], [16].

Here we employed a global GC–MS-based metabolite-profiling method using a validated approach [12] to measure levels of metabolites in the brain tissues of AD patients and controls. This study had two well-defined goals: to assess AD-associated metabolic disturbances of the brain; and to identify metabolites that may be targeted by new therapeutic approaches. A key outcome of this work resides in the finding that there are disease-specific metabolomic profiles that can be readily measured in the advanced stages of AD; it also points to the existence of cross-regional differences in metabolomic perturbations in advanced stages of the disease (Braak IV–VI).

## 2 Materials and methods

### 2.1 Acquisition of human brains

Whole brains from patients and matched controls were obtained from the New Zealand Neurological Foundation HBB, in the Centre for Brain Research, Faculty of Medical and Health Sciences, University of Auckland, Auckland, New Zealand. All procedures were approved by the University of Auckland Human Participants Ethics Committee with written informed consent from all families. The quality of AD-brain tissue acquired by the HBB was uniformly high, and only that with short post-mortem delay (~ 4–13 h) was used for the current study.

### 2.2 Sampling of human-brain tissue

After receipt into the HBB, brains were dissected under the supervision of neuroanatomists [17], to ensure accurate identification of each of the seven brain regions targeted in this study (HP, ENT, MTG, SCX, MCX, CG, and CB). Tissue samples of 50 ± 5 mg were dissected from each region and stored at − 80 °C until analysis.

### 2.3 Diagnosis and severity of AD

All AD patients had clinical dementia, whereas controls did not. Control brains were selected from the HBB by matching for age, sex and post-mortem delay (Table 1).

Details of individual patients, including cause(s) of death as certified by *post*-*mortem* examination, are summarized in Supplementary Table 1. A consultant neuropathologist diagnosed or excluded AD by applying the Consortium to Establish a Registry for AD (CERAD) criteria [18], and also determined the neuropathological severity by assigning a Braak stage [16] to each brain (Supplementary Table 1).

### 2.4 Tissue extraction

Brain tissues were placed in “Safe-Lok” microfuge tubes (Eppendorf AG; Hamburg, Germany) and held at − 80 °C until extraction. They then underwent a Folch-style extraction using a TissueLyser batch bead homogenizer (Qiagen; Manchester, UK). Briefly, each sample containing 50 ± 5 mg of brain tissue was extracted in 0.8 ml 50:50 (*v*/v) methanol:chloroform, to which a solution of the labeled internal standards in methanol had been added to achieve a final concentration of 0.016 mg/ml of each internal standard in the extraction solvent (kept at − 20 °C until used). A set of seven isotopically-labeled standards (citric acid-*d*_4_, ^13^C_6_-d-fructose, l-tryptophan-*d*_5_, l-alanine-*d*_7_, stearic acid-*d*_35_, benzoic acid-*d*_5_, and leucine-*d*_10_), purchased from Cambridge Isotopes Inc. (Tewksbury, MA), were used in this study. Extraction was performed for 10 min at 25 Hz with a single 3-mm tungsten carbide bead per tube. Samples corresponding to the same brain region were handled as single separate batches for this and all subsequent procedures. Separation of phases was achieved by addition of 0.4 ml of water followed by vortex-mixing (10–15 s) and centrifugation (2400 g, 15 min). After separation, tissue debris lay at the interface between the lower (non-polar, chloroform) phase and the upper (polar, methanol:water) phase containing the target molecules for the current study. For each batch, extraction blanks were prepared by processing tubes containing solvent and bead, but no tissue sample. This procedure produced clean polar extracts with low levels of lipid and protein content, which are known otherwise to cause response-instability in the GC–MS method.

### 2.5 Sample preparation

Chloroform in extraction tubes was removed using a 500-μl HPLC syringe (Sigma Aldrich, MO). Tubes were then centrifuged (16,000 *g*, 15 min) to encourage tissue debris to form a coherent pellet. From the methanol:water supernatant, 200-μl aliquots were transferred to pre-labeled tubes containing 600 μl of methanol, to precipitate residual protein. A quality-control (QC) pool was made by combining 200-μl aliquots from each extraction. The pooled samples were gently mixed and 200-μl portions dispensed into tubes containing 600 μl of methanol. Both sample and QC tubes were centrifuged (16,000 *g*, 15 min) and 750-μl aliquots were transferred to a final set of pre-labeled tubes which were processed to dryness in a Speedvac centrifugal concentrator (~ 30 °C, 16–18 h: Savant; SPD331DDA, Thermo Scientific). Dried residues were held in sealed tubes at 4 °C for up to one week (shown to be stable for eight weeks for serum previously stored) until derivatization for GC–MS analysis.

### 2.6 GC–MS analysis

Methyloxime/trimethylsilyl derivatives were prepared by a two-step procedure. GC–MS analysis was performed using an MPS2 autosampler (Gerstel; Mülheim an der Ruhr, Germany), a 7890A Gas Chromatograph with Split/Splitless inlet (Agilent; Santa Clara, CA), and a Pegasus HT time-of-flight mass spectrometer (LECO; Stockport, UK). The approach was based on our previously described method [12].

Gas chromatography was performed using an Agilent/J&W DB-17MS column (30 m × 0.25 mm × 0.25 μm; Agilent: #122–4732) with a 3-m deactivated Fused Silica retention gap (0.25 mm; Agilent: #No 160–2256-10), and helium carrier gas (1.4 ml/min, constant flow mode). 1-μl sample injections were made in Pulse Splitless mode at an inlet temperature of 270 °C, using an “empty, hot-needle” technique. The initial column temperature (50 °C) was held for 6 min and then increased to 300 °C at 10 °C/min and held for a further 4 min. This resulted in a total cycle time of 42 min between injections. After an initial 450-s solvent delay (to allow solvent and reagents to elute without damaging the detector), mass-spectral data were acquired at 10 spectra/s for the range 45–800 Da, detecting a range of amino acids, sugars, sugar alcohols and organic acids as their TMS derivatives. Standard 70 eV electron energy was employed, at a source temperature of 220 °C. Prior to sample analysis, the GC–MS was prepared for use as previously described [19].

The study was performed in a series of single-batch experiments, where each specific brain region constituted a batch. Within each batch, individual cases and controls were randomized, and run in a sequence interleaved with injections of the pooled QC samples (one per four study samples) and extraction blanks (two per batch). A lead-in sequence of six QC injections at the start of each batch was used to condition the chromatographic system. Extraction blanks were inspected visually to confirm absence of carryover, but not included in subsequent data analysis.

### 2.7 Data reduction

Data were prepared using the ‘Reference Compare’ method within ChromaTOF 4.5 (LECO). Briefly, the software was used to perform a global peak deconvolution of representative QC samples based on pre-defined parameters to compile a list of nominated ‘metabolites’, and search mass-spectral libraries to generate putative identities. Databases we employed were: the NIST Mass Spectral Reference Library (NIST08/2008; National Institute of Standards and Technology/Environmental Protection Agency/National Institutes of Health Spectral Library; NIST, Gaithersburg, MD); the Golm Metabolome Database (Max Planck Institute of Molecular Plant Physiology, Potsdam-Golm, Germany); and an in-house library developed at the University of Manchester [19]. Chromatographic retention-time data were available from reference-standard compounds for a subset of the identities. Within this subset, matching of both mass spectra and expected retention time(s) was interpreted to constitute a definitive (D) molecular identification. Matching of mass spectra and retention time with reported data was interpreted as confident (C) identification. Matching of mass spectra only was interpreted as a putative (P) identification.

**Fig. 1:**
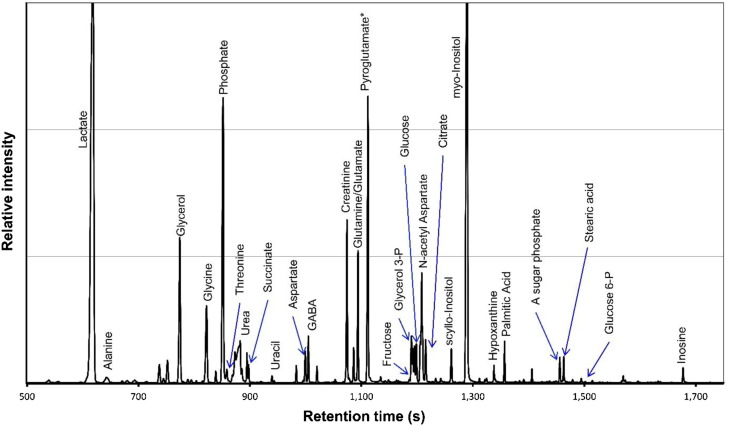
Shown is a GC–MS study of human-brain tissue showing a representative mass chromatogram of an extract of pooled entorhinal cortex. The y-axis has been expanded to allow visualization of lower-intensity peaks corresponding to the most abundant metabolites (hence the truncated off-scale peaks for lactate and myo-inositol). * Pyroglutamic acid is known to form from both glutamic acid and glutamine during derivatization for GC–MS.

From the list of nominations, we compiled reference tables comprising expected mass spectra and retention-time windows. These were then applied as target lists of features to be searched across all the study samples. As the pooled QC samples should contain all metabolite features encountered in the experiment, these were suitable candidates for compilation of reference tables. To provide a robust reference table, the initial list of nominations was edited to remove ambiguous and low-quality spectra prior to application. Global deconvolution was performed on several pooled QC injections across the entire experiment to improve identifications. By displaying these overlaid while editing the list, reproducible spectra were more readily distinguished from lower-quality candidates. The same target list was used for all brain regions. A representative chromatogram from the current experiment is shown in Fig. 1.

### 2.8 Metabolite abundance reporting

In order to use the edited reference table as a reporting tool, appropriate parameters such as mass-spectral match thresholds and tolerable retention-time deviations (6 s) were specified, and the table initialized using a pooled QC sample to provide reference m/z peak areas. Improved reproducibility was achieved by the use of internal standard ratios rather than raw peak areas. The most suitable standard was assigned to each metabolite by determining which internal standard yielded the lowest variance for a given metabolite across all the QC injections.

The resulting data for each experiment were compiled into a matrix of metabolite-intensity data, which was merged with experimental metadata for visualization and statistical analysis. Although the automated procedure was highly reliable (estimated return of correct peak areas for > 95% of features measured), data sets were also curated manually to remove possible integration errors which were mostly associated with metabolites showing non-ideal peak shape.

Analytical data for all the metabolites measured in this study have been included in Supplementary Table 2. Supplementary Table 3 presents data concerning metabolites which showed no statistically significant change in abundance in AD-brain.

### 2.9 Statistics

The merged metadata were used for data analysis. A principal-components analysis (PCA) was performed for visualization to confirm overall data integrity, using SIMCA-P software (UMetrics AB, Umeå, Sweden). Calculation of relative fold-change and statistical analysis were performed in log space using multiple *t*-tests (GraphPad Prism 6). Data were considered for multiple-comparison analysis by applying an FDR (10%) correction. The fold-changes were converted to linear space for presentation and metabolites identified in ≥ 5 samples in each group have been reported.

## 3 Results

**Fig. 2:**
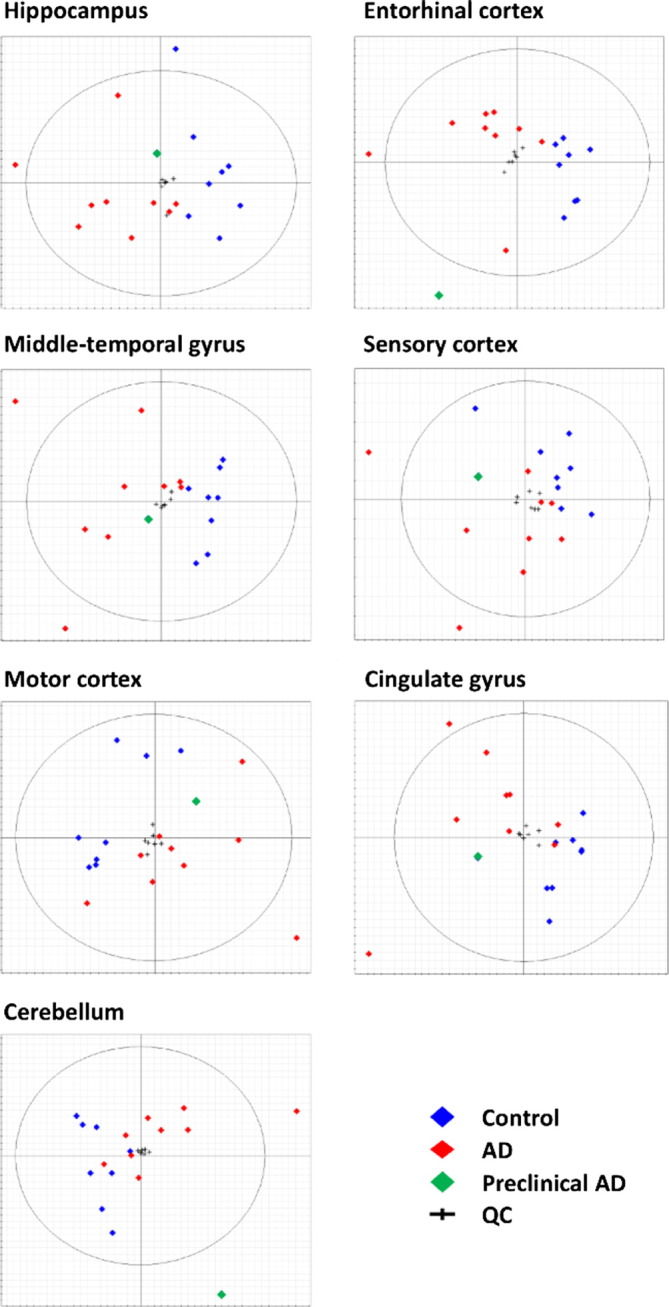
PCA-score biplots for seven brain regions showing class separations between control (*blue*) and AD (*red*) cases as demonstrated for each brain region. One control patient (*green*) had premanifest disease (Braak Stage II). Tight QC-clustering (crosses) in each brain region confirms low levels of technical variation throughout these measurements.

This study compared results from cases and controls with comparable age, sex, and *post*-*mortem* delay between study-groups. Median brain weight was ~ 16% lower in AD: median (range) brain weight was 1062 g (831–1355) in AD and 1260 g (1094–1461; *P* < 0.005) in controls (Table 1). PCA of GC–MS data revealed: 1) excellent class separation in all brain regions between AD and control samples; 2) greater biological than technical variation; and 3) absence of run-order effects (Fig. 2).

One control sample (*green* in Fig. 2) clustered more closely to the AD samples. The brain from which this sample originated had the lowest brain-weight (1094 g) among the controls and was assessed as Braak Stage II by neuropathological examination (individual No. 6, Supplementary Table 1). On this basis, this control sample was reclassified as a case of preclinical AD and has been excluded from the subsequent analysis for reasons of clarity.

**Fig. 3:**
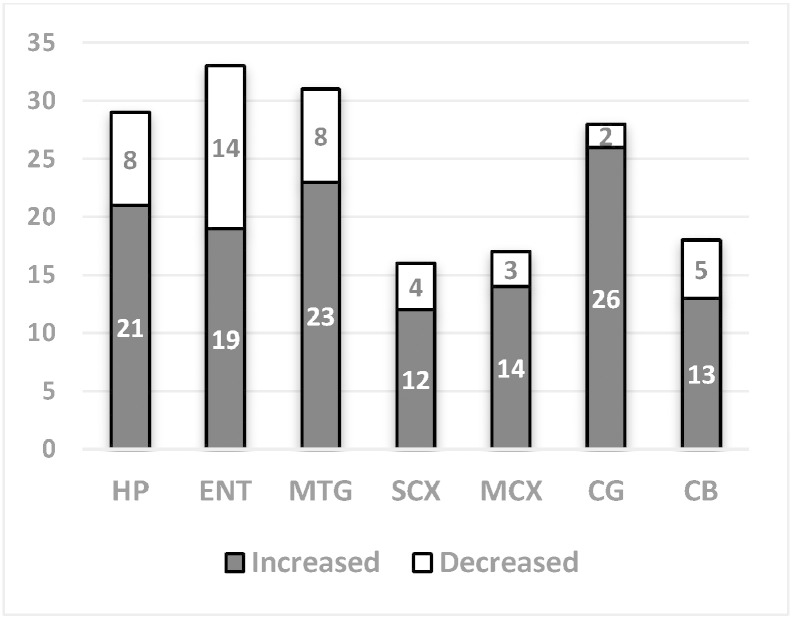
Number of metabolites altered in seven brain regions in AD cases compared with controls.

Untargeted GC–MS analysis enabled us to categorize 69 metabolite features per brain region (Table 2 and Supplementary Table 3). 55 features were shown to change in at least one brain region (FDR- corrected multiple *t*-test, *P* < 0.05), with individual regions showing between 16 and 33 metabolites identified as significantly changed (Fig. 3).

Those regions thought to be most heavily affected by AD according to neuropathological examination (HP, ENT, and MTG) and CG showed more metabolites to be significantly altered (28–33) compared to MCX and SCX (16–18). This observation may suggest a gradient of metabolic dysfunction associated with severity of damage. Surprisingly, CB (considered to be relatively spared in AD) exhibited similar levels of metabolite change to MCX and SCX (Fig. 3).

Specific findings of this study identify metabolites from several key biological pathways, including glucose utilization/clearance and brain energetics, and urea and amino-acid metabolism (Table 2).

Glycolysis begins with the conversion of glucose to glucose-6-phosphate (glucose-6P), followed by glucose-6P to fructose-6P. We found consistent elevation in both glucose and glucose-6P in all brain regions. Consistent with increased intracellular glucose levels, we also observed increased amounts of metabolites involved in alternative pathways for glucose metabolism: namely, the polyol pathway and the pentose–phosphate pathway. Glucose elevations trended higher in more severely-affected regions and were generally less marked, although still clearly elevated, in CB.

**Fig. 4:**
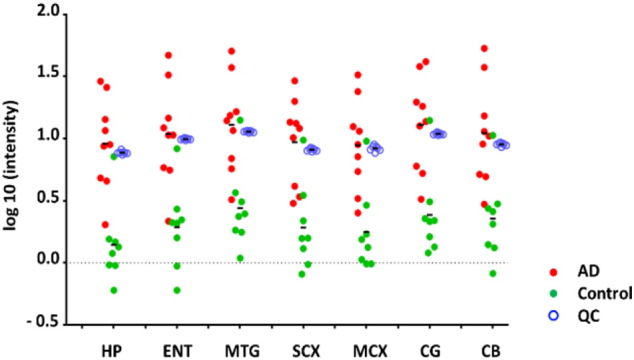
Urea levels in seven brain regions of AD and control brain as measured by GC/MS. The levels of urea were significantly higher (p < 0.05 in all studied regions) in AD cases (n = 9) compared to controls (n = 8). The tight grouping of QC values (*blue*, n = 7) indicate excellent reproducibility in the methodology. Points represent individual analyses.

To cope with its high energy demands, the brain is capable of switching to alternative fuel sources, including butanediol, β-hydroxybutyrate and lactic acid. These three metabolites were also significantly elevated in AD-brain, consistent with alternative fuel use. We also found increased levels of certain sugars (threitol, xylitol, and disaccharide not-otherwise-specified) and derivatives (N-acetylglucosamine, myo-inositol, and myo-inositol-1P). Glycerol levels were decreased, whereas the phosphate derivative, glycerol-3P, was increased (Table 2). Increases in fuels other than carbohydrates were less apparent in SCX and MCX. We found increased levels of two TCA cycle intermediates, citric acid and malic acid, in the heavily-affected regions of AD-brain. While the levels of urea were dramatically elevated in all brain regions (Fig. 4), the urea cycle metabolites ornithine and N-acetylglutamic acid were apparently decreased (Table 2).

Amino acids comprised the largest group of metabolites identified by this study to be altered in AD compared to control brain. There were no evident changes in the levels of branched-chain amino acids (Supplementary Table 3). Overall, there tended to be more significant alterations in the amino-acid levels in more severely affected brain regions. With respect to neurotransmitters and their precursors, 4-aminobutyric acid (GABA) was decreased in general while the aromatic amino acids (phenylalanine and tryptophan) were increased in AD-brain, most consistently in the ENT and MTG (Table 2).

Other significant findings include major decreases in levels of the nucleobases uracil and hypoxanthine, as well as ethanolamine in AD-brain. We also found increased levels of 2-hydroxyglutaric acid in AD-brain.

## 4 Discussion

This study has identified an extensive range of metabolic perturbations in AD-brain, which lead to a number of interesting possibilities. Metabolites are not only the building-blocks for biological components such as proteins and DNA, but are also central to intermediary metabolism which provides energy for cellular process, and for maintenance of structural integrity of tissues. Furthermore, metabolites can act as signaling molecules with regulatory functions in biological systems [19]. Therefore, systematic study of metabolites can provide important functional information concerning the status of a biological system. To the best of our knowledge, systematic multi-regional metabolite profiling of amino acids/neurotransmitters, sugars, sugar alcohols and organic acids has previously only been performed in biofluids in AD (for example, see references [20], [21], [22], [23], [24]) and this is the first GC–MS-based metabolite profiling carried out in human brain tissue in multiple brain regions from AD-cases and controls. The examination and comparison of seven functionally-distinct brain regions using this metabolic profiling approach is also unprecedented in the field of AD research.

Here, the close case–control matching and short *post-mortem* delays have contributed to the quality of our data (Table 1). The observed decrease in brain weight in AD is generally consistent with histological severity [16]. The included cases all had ‘classical’ or ‘usual’ AD as diagnosed clinically and by application of the CERAD and Braak criteria and are therefore representative of the sporadic form of the disease. Apart from the premanifest sample, which was excluded here from the final analysis, there were two AD samples (#14 and #16, see Supplementary Table 1) that appeared as outliers in the PCA plots (Fig. 1). However, no legitimate reasons for exclusion of the corresponding data were present in the patient history, and inclusion of these data did not result in qualitative change in the outcomes of this study: for example, neither the significance of the statistical analyses nor the fold-changes were affected.

Defective energy metabolism is a core component of AD pathology [25] and impaired brain-glucose uptake, known to manifest decades before the onset of clinical symptoms of AD, is believed to lie at the centre of this defect. AD-brains show impaired glucose uptake [26] and regional impairment of cerebral perfusion [15], which are thought to be consistent with low brain-glucose levels and cerebral hypometabolism being responsible for cognitive decline in AD [27].

Here, by contrast, we found robust evidence for marked, pan-cerebral *elevation* of free glucose in the AD-brain (Table 2), along with ubiquitous elevations in the levels of glucose-6P, sorbitol and fructose: these findings are consistent with impaired glucose utilization via glycolysis coupled to enhancement of alternate pathways of carbohydrate metabolism, for example the polyol pathway and the pentose–phosphate pathway. The localized accumulation of fructose-6P in MTG and CG points to a block in glycolysis distal to this metabolite in these tissues. Concomitant elevations in both free fructose and fructose-6P could possibly be linked via the action of ketohexokinase (fructokinase; E.C. 2.7.1.4) which is known to be expressed in nervous tissue, but this hypothesis will require experimental testing. The elevations in free glucose and fructose in the AD-brain tissues measured here have the potential to exert toxigenic effects, consistent with their actions in diabetes mellitus, where they are clearly responsible for organ damage (for example, in the heart, arteries, kidneys, peripheral nerves, and retina). These observations are also consistent with reports of decreased gene expression of glycolytic enzymes in the HP of AD-brain [28], [29].

Elevated erythronic acid, previously identified as a major hallmark of pentose–phosphate pathway defects [30], is further consistent with abnormal pentose–phosphate pathway function in certain regions of AD-brain. This finding is consistent with previously reported up-regulation of the pentose–phosphate pathway in mild cognitive impairment (MCI) that later progressed to AD [22].

The use of alternative fuel sources (other than glucose) is critical for energy production in the brain during starvation, or when glucose utilization is impaired, as in AD [31]. Apart from the principal energy-generating substrate, glucose, the brain is also capable of utilizing alternative substrates such as fatty acids and ketone bodies [32]. Elevated levels of the principal ketone body and its precursor (β-hydroxybutyrate and butanediol) are consistent with a general elevation in the availability of these substrates in the AD-brain. Marked elevation in 2-hydroxy-3-methylbutyric acid, a marker for lactic acidosis and diabetic keto-acidosis [33], is further consistent with increased metabolism of ketone bodies in AD. The elevation of numerous sugars other than glucose, and their derivatives, most significantly threitol and myo-inositol-1P, may represent either a compensatory mechanism for defective energetics (as in the case of ketone bodies) or occur as a result of defective metabolism of these substrates in the AD-brain, as suggested to be the case for glucose.

Degradation of phospholipids and triglycerides releases fatty acids and glycerol, the latter of which can be converted to glucose via the intermediate, glycerol-3P. Decreased glycerol with concomitantly increased glycerol-3P (as well as glucose) in the HP and ENT may suggest that conversion of glycerol to glucose is favored in these regions of the AD-brain (perhaps again associated with the need for extra energy sources).

Glycolysis coupled with the TCA cycle constitutes the major pathway of energy generation in the brain [34]. As the final common pathway for substrates such as carbohydrates and fatty acids, which can be transformed into acetyl groups, the TCA cycle is critical for ATP production. The observed accumulation of TCA-cycle intermediates in heavily affected brain regions (HP, ENT, and MTG) in this study is in line with reported TCA-cycle abnormalities, characterized by decreased activity of key enzymes, in AD [35].

The urea cycle is critical for maintaining ammonia and amine-nitrogen homeostasis through its role in amino-acid metabolism, and impaired urea-cycle activity can lead to hyperammonemia, a major component of certain classes of acute neurological disturbances [36].

It has been asserted that there is no functional urea cycle in the brain, and little available evidence for the existence of a functional urea cycle in the brain has been published to date. However, we recently ascertained by data-base searching, that there is substantive evidence that all the enzyme-components of the urea cycle are transcribed in the brain tissue, and that all but two of these are also translated into protein there (data not shown). Data-bases employed for these studies were as follows: mRNA: BioGPS, GTEx, CGAP, and TAG; and proteomics: Proteomics DB, Pax Db, MOPED, and MaxQB. Urea-cycle enzymes for which proteome-level evidence for brain expression is currently lacking to our knowledge are ornithine carbamoyltransferase (EC 2.1.3.3) and arginase 2 (EC 3.5.3.1): this lack of evidence could reflect low expression levels and may not constitute evidence of absence. We conclude that the potential presence of urea cycle activity in brain tissue seems not to have been systematically excluded to date and that, accordingly, the potential exists that a functional urea cycle, capable of generating urea from suitable substrates, could be operative in brain tissue, for example in astrocytes. Further direct experimental evidence for the potential presence of the urea cycle in brain tissue and in astrocytes, will need to be sought in future experiments.

Here, one of the most striking changes observed in AD-brain was the marked elevation of urea levels across all regions examined (Table 2, Fig. 4). By contrast, systemic over-production of urea, leading to elevated urea levels, for example, in the plasma, is not known to occur in AD. Urea is generally regarded as a detoxification product formed from ammonia/ammonium ion and/or amine-nitrogen moieties. However, urea itself can also be toxic at sufficiently elevated levels, according to systematic studies of the impact of elevated urea levels in cell-culture and in vivo rodent models [37]. Our current findings are consistent with impaired local urea regulation in brain in AD, by up-regulation of its synthesis and/or defective clearance.

We hypothesize that defective urea metabolism could play a substantive role in the pathogenesis of neurodegeneration in AD, perhaps via defects in osmoregulation or nitrogen metabolism that lead to or cause toxigenic accumulation of urea in the brain. This finding is of potential importance, since it indicates that intervention aimed at lowering of brain-urea levels, possibly in combination with interventions targeted at improving cerebral carbohydrate metabolism, could provide a potential new therapeutic strategy for AD.

Nitrogen derived from amino-acid catabolism can enter the urea cycle in the form of ammonium ions or glutamate, via transdeamination or transamination routes. In the transdeamination route, α-ketoglutarate accepts an amino group from the donor amino acid to form glutamate, the deamination of which in turn generates ammonium ion for incorporation into carbamoyl phosphate, which then reacts with ornithine to enter the urea cycle as citrulline. The formation of carbamoyl phosphate is catalyzed by carbamoyl-phosphate synthase 1 (EC 6.3.4.16) in the presence of its allosteric effector, N-acetylglutamic acid.

Here, the observed decrease in both ornithine and N-acetylglutamic acid and concomitant increase in urea, suggests that mechanisms other than altered urea cycle activity could also contribute to the observed brain-urea build-up. Other sources of urea may be from detoxification of ammonia through the glutamine cycle in astrocytes, and by transaminase-catalyzed reactions in neurons and astrocytes involving alanine, aspartate and glutamate. These reactions could alter the glutamate/glutamine balance, which could help explain the elevated glutamate levels seen in CG in this study. We note that other reports of changes in glutamate levels in the AD-brain are contradictory, possibly due to general differences in the measurements performed in *ante*-*mortem* and *post-mortem* tissue [38], [39].

An alternative route of glutamate metabolism is via transamination, which involves two linked transamination reactions. The first step is identical to the transdeamination route and the second step involves oxaloacetate, the oxidized derivative of malate (increased in HP), which accepts an amino group from glutamate to form aspartate. Elevated ammonia concentrations can impair the malate–aspartate shuttle [40], [41] so the significant decrease in aspartate (here observed in HP and ENT) is consistent with impairment of the transamination route, possibly as a result of hyperammonemia in the HP. Consistent with our finding, elevated urea and ammonia levels have previously been reported in CSF from AD patients [42].

The change in amino-acid levels in the AD-brain appeared to be more closely localized to heavily-affected brain regions. While this finding is consistent with a previously-proposed association between impaired metabolic status and amino-acid levels in AD-brain [43], our findings are further consistent with a gradient of severity across different brain regions.

Amino acids also play a critical role in the brain through their role as neurotransmitters and their precursors. Glutamate is not only the main excitatory neurotransmitter; it is also the precursor of GABA, an inhibitory neurotransmitter. Our current findings of increased glutamate and decreased GABA levels are consistent with a previously-reported decrease in glutamate decarboxylase 1 (GAD1 brain; EC 4.1.1.15), the enzyme responsible for conversion of glutamate to GABA in the AD-brain [44], and increased glutamate in the CSF of patients with AD [42], [45]. Gamma-hydroxybutyrate (GHB) is formed primarily from GABA by cerebral neurons [46] and hence decreased GHB is likely a consequence of decreased GABA.

Aromatic amino acids are precursors for the monoamine (serotonin) and catecholamine (dopamine, norepinephrine, and epinephrine) cerebral neurotransmitters, whose biosynthesis is sensitive to local substrate concentrations [47]. The observed increase in the levels of aromatic amino acids (phenylalanine and tryptophan) may be associated with previously-reported neurotransmitter imbalance, as exemplified by lowered levels of serotonin [48], [49], dopamine [49], and norepinephrine [50] in AD-brain. These findings are also in line with the proposed effect of hyperammonemia (resulting from urea cycle impairment) on the supply of neurotransmitter precursors (e.g. tryptophan) across the blood–brain barrier, which alters the cerebral synthesis and catabolism of neurotransmitters (e.g. serotonin) [36].

Previously, increased levels of phenylalanine have been reported in the CSF of AD patients [51]. The altered levels of neurotransmitter precursors found in this study are consistent with either defective neurotransmitter synthesis or elevated neurotransmitter degradation in the AD-brain.

Genomic stability is particularly important in the brain, as neurons are terminally differentiated and have high metabolic activity resulting in the generation of large amounts of reactive oxygen species [52]. A balance in the level of nucleobases/nucleosides is critical for genomic stability. In this study, levels of the nucleobase adenine, the nucleoside guanosine, and the nucleotide adenosine-5-monophosphate were increased (where statistically significant) in regions of the AD-brain. Interestingly, the catabolic intermediate of purine metabolism, hypoxanthine was decreased significantly in the AD-brain. Another marked change observed in this study was the decrease in the nucleobase uracil. The direct effect of lowered uracil levels in the brain is unknown. However, impaired repair of uracil residues induced by folate deficiency has been suggested to participate in neurodegeneration [53].

Additional changes in metabolites that may also play important roles in the pathology of AD include ethanolamine, the second-most abundant head group in phospholipids, whose levels were decreased in all brain regions in this study. This finding could be associated with altered phospholipid levels in the AD-brain. Elevated 2-hydroxyglutaric acid levels may also be highly relevant in AD, considering its potential detrimental role in the central nervous system, for example as reported in a patient with hydroxyglutaric aciduria [54].

In summary, the present study has identified widespread metabolic perturbations in the human AD-brain, including those regions that have hitherto been considered to be less affected or relatively spared in the disease process. These metabolic perturbations are of particular interest as they represent molecular changes that can occur prior to the volumetric loss, for example in brain regions such as sensory and motor cortices, and CB, and are therefore likely to comprise an important part of the early pathogenesis of AD. Several of the identified perturbations have the potential to exert toxic functions (for example, elevated brain-urea and brain-glucose levels) and could therefore contribute individually or in sum to the pathogenesis of neurodegeneration and dementia in patients. One or more of these perturbations could serve as a potential target for novel therapeutic interventions in AD.

The following are the supplementary data related to this article.

Supplementary Table 1 Individual patient characteristics. Supplementary Table 1

Supplementary Table 2 Analytical information of metabolites. Supplementary Table 2.

Supplementary Table 3 Metabolites with no statistically significant change in abundance in the AD brain. Changes in metabolites were shown in fold-change, AD/controls. Supplementary Table 3

## Author contributions

JX designed and performed research, analyzed and interpreted data, and wrote the manuscript; PB designed and performed research, analyzed data, and revised the manuscript; SJC, SP, KAH, MJ, HJW, MAC, RLMF and RDU performed research, analyzed data and revised the manuscript; GJSC conceived, designed and supervised research, analyzed and interpreted data, wrote the manuscript, and bears overall responsibility for the integrity of the study and of the manuscript.

## Funding

This work was supported by the Endocore Research Trust [61047]; the Maurice and Phyllis Paykel Trust [3627036; and Travel funding for JX]; Lottery Health New Zealand [3626585; 3702766]; the Maurice Wilkins Centre for Molecular Biodiscovery [through a Tertiary Education Commission grant 9341-3622506; and a Doctoral Scholarship for JX]; the Health Research Council of New Zealand [3338701]; the University of Auckland [Doctoral Student PReSS funding JXU058]; the Oakley Mental Health Research Foundation [3456030; 3627092; 3701339; 3703253; and 3702870]; the Ministry of Business, Innovation & Employment [UOAX0815]; the New Zealand Neurological Foundation; the Medical Research Council [UK; MR/L010445/1 and MR/L011093/1]; Alzheimer's Research UK (ARUK-PPG2014B-7); the University of Manchester, the Central Manchester (NHS) Foundation Trust, and the Northwest Regional Development Agency through a combined program grant to CADET; and was facilitated by the Manchester Biomedical Research Centre and the Greater Manchester Comprehensive Local Research Network.

## Conflict of interest statement

The authors declare that they have no conflict of interest with respect to this work. Sponsors had no role in the study design; the collection, analysis and interpretation of data; the writing of the manuscript; or the decision to submit the article for publication.

## Transparency document

Transparency document. Image 1

## Figures and Tables

**Table 1:** Group characteristics.

Variable	Control	AD
Number	9	9
Age (± SD)	70.1 (± 6.7)	70.3 (± 7.1)
Male sex, n (%)	5 (55.6)	5 (55.6)
PMD (h)	9 (5.5–13.0)	7 (4.0–12.0)
Brain weight (g)	1260	1062*
(1094–1461)	(831–1355)

Values are: age, mean (SD); *post*-*mortem* delay (PMD) and brain weights, median (range): *P = 0.005 compared with Control; all other differences were non-significant

**Table 2:** Metabolites with altered abundance in AD-brain tissue. Numbers indicate fold-changes (AD/controls). Changes with *P* < 0.05 (10% FDR) were considered significant and are shown here in ***bold italic font***.

Metabolite	HP	ENT	MTG	SCX	MCX	CG	CB
*Glucose and related metabolites & pentose phosphate pathway components*							
Glucose (D)	***12***.***3***	***9***.***3***	***16***.***9***	***10***.***8***	***8***.***0***	***9***.***9***	***6***.***8***
Glucose-6-phosphate (D)	***5***.***9***	***4***.***9***	***8***.***5***	***4***.***8***	***6***.***0***	***4***.***8***	***3***.***8***
Sorbitol (D)	***3***.***4***	***3***.***8***	***3***.***9***	***5***.***0***	***5***.***3***	***5***.***0***	***4***.***6***
Fructose (D)	***4***.***6***	***4***.***8***	***7***.***0***	***6***.***8***	***7***.***0***	***7***.***0***	***7***.***1***
Fructose-6-phosphate (D)	1.2	0.6	***5***.***3***	1.6	2.5	***7***.***7***	2.3
Pentonic acid A (P)	1.1	***1***.***3***	***1***.***3***	1.2	1.4	***1***.***4***	1.3
Pentonic acid B (P)	***2***.***1***	***2***.***0***	***1***.***9***	***1***.***7***	***1***.***7***	***1***.***9***	1.7
Arabinose (P)	***3***.***5***	NM	***3***.***6***	***3***.***5***	***6***.***8***	***3***.***9***	NM
Ribose-5-phosphate (D)	0.8	***0***.***6***	***0***.***8***	1.1	1.1	1.0	0.9
Erythronic acid (P)	***1***.***6***	***1***.***8***	1.1	1.2	1.2	1.4	1.5

*Alternative fuel sources*							
Butanediol (D)	4.2	***4***.***3***	1.6	***4***.***2***	***9***.***3***	***4***.***1***	***4***.***1***
β-Hydroxybutyric acid (D)	1.6	2.5	1.5	***3***.***4***	1.8	***2***.***5***	1.6
Lactic acid (D)	***2***.***6***	0.5	1.3	3.4	7.3	1.7	0.6
2-hydroxy-3-methylbutyric acid (P)	***7***.***7***	2.3	2.8	***4***.***3***	2.5	***4***.***7***	3.4
Threitol (D)	***2***.***0***	***2***.***3***	***1***.***8***	***2***.***4***	***2***.***3***	***2***.***4***	***3***.***2***
Xylitol (D)	***1***.***7***	1.3	1.2	1.2	1.1	***1***.***4***	1.7
Disaccharide (D)	***4***.***8***	2.3	***3***.***2***	3.6	1.8	***1***.***8***	0.9
N-acetylglucosamine (C)	1.2	1.1	***1***.***7***	1.4	1.4	***1***.***6***	2.8
Myo-inositol (D)	1.1	***1***.***9***	1.1	1.2	0.8	0.7	0.9
Myo-inositol-1-phosphate (P)	***2***.***3***	***2***.***0***	***4***.***1***	1.7	***2***.***2***	***3***.***9***	***3***.***5***
Glycerol (D)	***0***.***6***	***0***.***7***	0.8	0.8	0.7	0.9	0.9
Glycerol-2-phosphate (P)	***1***.***9***	***1***.***6***	***1***.***5***	1.7	1.8	1.***6***	***1***.***6***
Glycerol-3-phosphate (D)	***2***.***3***	***2***.***7***	1.4	***2***.***5***	***2***.***6***	***1***.***8***	***1***.***6***
Glyceric acid (P)	1.3	1.1	***2***.***8***	1.5	1.1	***2***.***6***	***1***.***8***

*TCA cycle & urea cycle*							
Citric acid (D)	***1***.***7***	***2***.***1***	1.7	1.9	1.1	1.1	1.1
Malic acid (C)	1.6	***1***.***9***	***2***.***4***	1	0.8	1.7	0.9
Fumaric acid (C)	1.8	1.3	1.7	1.2	0.8	***1***.***4***	1.3
Ornithine (D)	***0***.***6***	***0***.***6***	1.0	0.7	0.7	0.9	***0***.***3***
Urea (D)	***6***.***5***	***5***.***6***	***4***.***7***	***4***.***9***	***5***.***0***	***5***.***3***	***4***.***9***
N-acetylglutamic acid (D)	0.4	***0***.***8***	0.9	0.8	0.7	0.8	1.0
Creatinine (D)	1.1	1.0	***1***.***5***	1.0	1.0	1.2	***1***.***2***

*Amino acids*							
Proline (D)	***0***.***5***	***0***.***4***	0.5	0.7	0.8	0.8	0.5
Lysine (D)	***0***.***5***	0.7	1.1	0.8	1.1	1.1	0.3
Glycine (D)	0.9	***0***.***7***	***0***.***8***	0.8	0.7	0.9	0.9
Serine (D)	***0***.***7***	***0***.***6***	1.0	0.7	0.8	1.0	***0***.***7***
Threonine (D)	0.8	1.3	***1***.***5***	1.1	2.2	1.4	1.7
Cysteine (D)	***2***.***0***	1.4	0.7	1.0	1.0	***1***.***4***	0.9
beta-Alanine (D)	1.2	1.0	***1***.***3***	1.1	1.1	1.3	0.9
Aspartic acid (D)	***0***.***6***	***0***.***6***	0.8	1.0	0.9	0.9	0.8
N-acetylaspartic acid (D)	0.7	0.8	***0***.***7***	1.0	0.9	0.9	1.0
Glutamic acid (P)	1.3	1.0	1.0	1.3	1.3	***1***.***3***	2.6
GABA (D)	1.3	***0***.***6***	***0***.***5***	***0***.***7***	0.7	0.9	0.8
4-hydroxybutyric acid (C)	0.6	***0***.***6***	1.1	0.8	0.8	0.7	0.7
Phenylalanine (D)	1.3	1.2	***2***.***1***	1.6	1.8	***2***.***0***	1.3
Tryptophan (D)	***2***.***5***	***2***.***2***	***4***.***7***	1.8	***3***.***2***	***4***.***0***	1.1

*Nucleosides*							
Adenine (D)	1.0	1.0	0.9	1.0	0.9	0.9	***1***.***7***
Uracil (C)	***0***.***6***	***0***.***5***	***0***.***6***	***0***.***6***	***0***.***5***	***0***.***6***	***0***.***7***
Adenosine-5-monophosphate (P)	1.6	***2***.***1***	1.9	1.4	NM	2.4	1.3
Guanosine (D)	0.7	0.8	***2***.***8***	0.9	NM	2.5	1.6
Hypoxanthine (D)	0.7	***0***.***7***	***0***.***7***	***0***.***7***	0.7	0.8	***0***.***7***

*Miscellaneous*							
Ethanolamine (D)	***0***.***6***	***0***.***4***	***0***.***5***	***0***.***5***	***0***.***5***	***0***.***6***	***0***.***6***
Methyl-phosphate (C)	0.7	***0***.***4***	***0***.***4***	0.7	0.6	0.5	0.8
Phosphoric acid (D)	1.2	1.1	0.5	0.8	***0***.***7***	0.6	0.9
2-Hydroxyglutaric acid (D)	***1***.***9***	***2***.***2***	***1***.***9***	1.6	***1***.***6***	***1***.***8***	1.4
Ascorbic acid (P)	***2***.***0***	1.7	1.1	1.8	***2***.***2***	1.6	1.5

Abbreviations: D, definitive; C, confident; P, putative.
