# Supplementary material for: Graded perturbations of metabolism in multiple regions of human brain in Alzheimer's disease: Snapshot of a pervasive metabolic disorder
Source: Biochim Biophys Acta. 2016 Jun;1862(6):1084–92. doi: 10.1016/j.bbadis.2016.03.001 (PMC4856736; doi:10.1016/j.bbadis.2016.03.001)
Supplement: Supplementary Table 1 — Individual patient characteristics. [file mmc1.docx]

| **Supplementary Table 1**  **Individual patient characteristics** | | | | | | | | | |
| --- | --- | --- | --- | --- | --- | --- | --- | --- | --- |
| No | Code | Group | Age/Sex | | *Ante-mortem* brain disease/mental state | Cause of death | Braak Stage | PMD (h) | Brain Wt (g) |
| 1 | H155 | Control | | 61/M | No brain disease or dementia | Ischemic heart disease | 0 | 7.0 | 1258 |
| 2 | H121 | Control | | 64/F | No brain disease or dementia | Pulmonary embolism | 0 | 5.5 | 1260 |
| 3 | H132 | Control | | 63/F | No brain disease or dementia | Ruptured aorta | 0 | 12.0 | 1280 |
| 4 | H122 | Control | | 72/F | No brain disease or dementia | Emphysema | 0 | 9.0 | 1230 |
| 5 | H204 | Control | | 66/M | No brain disease or dementia | Ischemic heart disease | 0 | 9.0 | 1461 |
| 6 | H241 | Control | | 76/F | No brain disease or dementia | Metastatic carcinoma | II | 12.0 | 1094 |
| 7 | H164 | Control | | 73/M | No brain disease or dementia | Ischemic heart disease | 0 | 13.0 | 1315 |
| 8 | H123 | Control | | 78/M | No brain disease or dementia | Ruptured aortic aneurysm | 0 | 7.5 | 1260 |
| 9 | H150 | Control | | 78/M | No brain disease or dementia | Ruptured MI | 0 | 12.0 | 1416 |
| 10 | AZ42 | AD | | 60/M | Alzheimer’s dementia | Alzheimer’s disease | VI | 7.0 | 1020 |
| 11 | AZ71 | AD | | 62/F | Alzheimer’s dementia | Alzheimer’s disease | VI | 6.0 | 831 |
| 12 | AZ48 | AD | | 63/F | Alzheimer’s dementia | Bronchopneumonia | VI | 7.0 | 1080 |
| 13 | AZ72 | AD | | 70/F | Alzheimer’s dementia | Lung cancer | V | 7.0 | 1044 |
| 14 | AZ90 | AD | | 73/M | Alzheimer’s dementia | GI hemorrhage | IV | 4.0 | 1287 |
| 15 | AZ96 | AD | | 74/F | Alzheimer’s dementia | Metastatic cancer | V | 8.5 | 1062 |
| 16 | AZ39 | AD | | 74/M | Alzheimer’s dementia | Pseudomonas bacteremia | VI | 12.0 | 1355 |
| 17 | AZ80 | AD | | 77/M | Alzheimer’s dementia | Myocardial infarction | VI | 4.5 | 1180 |
| 18 | AZ38 | AD | | 80/M | Alzheimer’s dementia | Bronchopneumonia/ pulmonary edema | V | 5.5 | 1039 |
| **Abbreviations**: GI, gastrointestinal; MI, myocardial infarction; PMD, post-mortem delay; wt, weight. Cause of death was determined by post-mortem examination, and brain pathology and Braak Stage were determined by specialist neuropathological examination. Causes of death were the primary causes listed on the death certificate. Patient H241 was found to have post-mortem signs consistent with AD and was therefore diagnosed with prodromal disease: the corresponding data have been retained in the main analysis presented in the manuscript, and removed from the control group for the secondary analysis. | | | | | | | | | |
