# Supplementary material for: Graded perturbations of metabolism in multiple regions of human brain in Alzheimer's disease: Snapshot of a pervasive metabolic disorder
Source: Biochim Biophys Acta. 2016 Jun;1862(6):1084–92. doi: 10.1016/j.bbadis.2016.03.001 (PMC4856736; doi:10.1016/j.bbadis.2016.03.001)
Supplement: Supplementary Table 3 — Metabolites with no statistically significant change in abundance in the AD brain. Changes in metabolites were shown in fold-change, AD/controls. [file mmc3.docx]

| **Supplementary Table 3**  **Metabolites with no statistically significant change in abundance in the AD brain**. Changes in metabolites were shown in fold-change, AD/controls. | | | | | | | |
| --- | --- | --- | --- | --- | --- | --- | --- |
| **Metabolite** | **HP** | **ENT** | **MTG** | **SCx** | **MCx** | **CG** | **CB** |
| Hydroxylamine (P) | 1.0 | 1.1 | 0.8 | 1.0 | 1.2 | 0.9 | 1.2 |
| Alanine (D) | 0.8 | 0.7 | 1.3 | 0.6 | 3.4 | 1.0 | 1.2 |
| Pyruvic acid (D) | 1.3 | 1.6 | 1.5 | 0.8 | 0.5 | 1.0 | 0.8 |
| Valine (D) | 1.6 | 0.9 | 2.8 | 1.0 | 1.0 | 1.5 | 0.5 |
| Leucine (D) | 0.9 | 0.9 | 1.4 | 1.0 | 1.0 | 1.4 | 0.8 |
| Isoleucine (D) | 0.8 | 0.8 | 1.3 | 0.8 | 1.0 | 0.7 | 1.0 |
| Succinic acid (C) | 1.3 | 0.6 | 1.1 | 1.2 | 1.3 | 1.2 | 1.2 |
| Methionine (D) | 1.0 | 0.9 | 1.3 | 0.9 | 1.0 | 1.4 | 0.7 |
| Ribitol (P) | 0.3 | 1.0 | 2.0 | 0.4 | 1.2 | 1.1 | 1.0 |
| Pyroglutamic acid (D) | 1.2 | 1.2 | 1.1 | 1.3 | 1.5 | 1.2 | 1.1 |
| Mannitol (D) | 1.1 | 1.2 | 1.3 | 1.3 | 1.2 | 1.2 | 1.8 |
| Scyllo-inositol (D) | 0.9 | 0.9 | 1.1 | 0.7 | 0.7 | 0.8 | 0.7 |
| Tyrosine (D) | 0.7 | 0.7 | 1.2 | 1.0 | 1.0 | 1.2 | 0.6 |
| Adenosine (D) | 2.0 | 1.3 | 0.9 | 1.0 | 1.0 | 0.8 | 1.0 |
| Abbreviations: D, definitive; C, confident; P, putative. | | | | | | | |
